# Biobased Oligo‐ and Polyesters from Fatty Acids with Demonstrated Biodegradability or Recyclability for Industrial Applications in a Circular Economy

**DOI:** 10.1002/cssc.70809

**Published:** 2026-08-27

**Authors:** Harald Gröger

**Affiliations:** ^1^ Chair of Industrial Organic Chemistry and Biotechnology, Faculty of Chemistry Bielefeld University Bielefeld Germany

**Keywords:** biodegradable chemicals, biorenewables, fatty acids, lubricants, polyesters, polyethylene‐like plastics, polymers

## Abstract

This “Perspective”‐article describes and discusses two recent developments in the field of biobased and biodegradable or chemically recyclable oligomers/polymers for applications in the lubricant and plastics product segments, which are accessible from unsaturated fatty acids as feedstocks. Within an industrial–academic collaboration, oleic acid was converted into hydroxymethylated stearates using the matured industrial technologies hydroformylation and hydrogenation. Subsequent solvent‐free enzymatic esterification then furnished a novel generation of lubricants that are biobased, ensure rapid biodegradation and show needed performance data. Furthermore, when starting from a C18 diester being also derived from fatty acid‐based sources, polyesters were designed through polycondensation with biobased diols such as C18 diol or ethylene glycol in the presence of a titanium alkoxide catalyst. The resulting polyesters showed material properties being comparable to high density polyethylene‐type materials and enabled at the same time a closed‐loop recycling due to their proven degradability properties. Both case studies illustrate the need to integrate various research issues “along the value chain” in such a product design, which range from biorenewable raw material selection, choice of economically attractive monomer synthesis, efficient polymerization steps and product features covering performance as well as sustainability properties such as biodegradability or (molecular) recyclability.

## Graphical abstract

This “Perspectives”‐article describes and discusses two recent developments in the field of biobased as well as biodegradable or chemically recyclable oligomers/polymers for applications in the lubricant and plastics product segments, which are efficiently accessible and which are based on the utilization of unsaturated fatty acids as feedstocks.

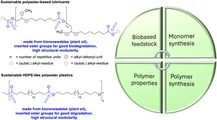

## 1 Introduction

Among major challenges for a circular economy is the design of novel industrial mass products that are not only made from biorenewable instead of fossil feedstocks but also fulfill the criterion of biodegradability [1, 2, 3]. While not for all applications biodegradability is desired (e.g., for products used in the construction sector with the need of long durability), for a range of applications biodegradability is or will increasingly be a mandatory property. This is the case in particular for those applications with direct contact of such chemicals with the natural environment leading to a contamination by such products.

Lubricants are a class of products which “by definition” will come into contact with the natural environment, at least when being used for outdoor “mobility applications” such as, e.g., for cars, trucks, or ships. At the same time, lubricants are urgently needed due to their beneficial impact on energy saving. It has been calculated that lubricant solutions contribute to reduction of friction to an enormously impressive extent, leading to annual energy savings of up to 20 million terajoules worldwide [4]. This represents 3% of the total global energy consumption taking into account a global total energy demand of 650 exajoules [5]. Lubricants are produced on large scale, and already in 2015 their production volume exceeded 35 million tons. However, such lubricant molecules are mainly based on fossil feedstocks and most of them are not biodegradable yet or only biodegradable with difficulties, which usually leads to severe environmental pollution. Accordingly, the availability of biobased and biodegradable lubricants would have an enormous impact on environmental protection and represents a major current industrial challenge.

Another example for an industrial mass product that, however, enters the environment not by definition but by inappropriate disposal are plastics such as plastics bags [6]. Often such materials, which are made to a large extent from polyethylene (PE), remain in the environment for a long time due to the lack of suitable biodegradation properties. Consequently, also for such products the development of biobased and biodegradable or molecular recyclable alternatives while keeping the desired properties and enabling their production at low cost remained as a task of utmost importance.

**FIGURE 1:**
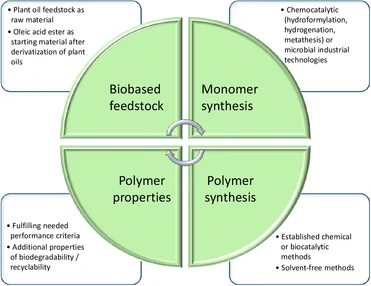
Overall concept and criteria for preparing biobased and biodegradable lubricants and recyclable plastics from oleic acid‐derived plant oil feedstocks.

In the following “Perspective”‐article, two recent developments in this area of biobased and biodegradable oligomers and polymers for applications in the lubricant and plastics product segments, respectively, are summarized and discussed. Both chosen case studies are based on the utilization of fatty acids as feedstocks and the introduction of a second functionality to obtain a monomer for oligo‐ and polyester formation in a condensation process. On the one hand, these representative examples should illustrate the importance of designing monomers that can be produced by means of technically feasible chemocatalytic or microbial production technologies at low costs as a prerequisite for commodity chemicals. In addition, these examples demonstrate how such economically attractive biobased monomers can then be converted efficiently into oligo‐ and polyesters, which not only fulfill the required criteria for the desired applications but also show unique sustainability properties such as biodegradability (in case of lubricants and plastics) or molecular recyclability (for plastics applications). The need to integrate research issues in such a product design, which range from biorenewable raw material selection, choice of economically attractive monomer synthesis, efficient polymerization steps, and product features covering oligomer/polymer properties as well as sustainability needs such as biodegradability or (molecular) recyclability, are summarized in Figure 1.

## 2 Design of Biobased & Biodegradable Lubricants and Process Development for Their Synthesis

**SCHEME 1:**
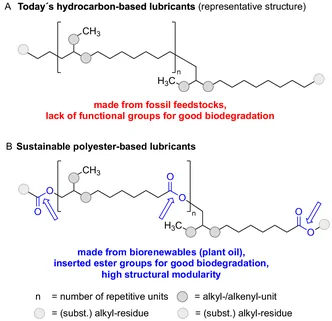
Conceptual design of biodegradable and biobased lubricant alternatives to fossil feedstock‐based lubricants, which still contain the branched structures favored for lubricants.

To start with the industrial segment of biobased and biodegradable lubricants, a strong driving force for the development of such products also came from regulatory changes. For example, in the United States a new “General Vessel Permit” (GVP) was taken into action in December 2013, which requested to use biodegradable oils, so‐called “Environmentally Acceptable Lubricants (EAL)”, at all oil‐sea‐interfaces if technically feasible [7]. This GVP regulation then stimulated innovation activities to design and develop such biodegradable lubricants. Teaming up with the international lubrication company Klüber Lubrication München (Germany), the initial steps of joint research activities with our research group consisted in a conceptual design of structures, which were expected to ensure the needed technical performance but also fulfill the criteria of biodegradability, biobased origin and economic production (Scheme 1) [8, 9].

When analyzing typical current industrial lubricants that are pure hydrocarbons (schematically illustrated by a representative model structure in Scheme 1A), it became evident that functional groups have to be introduced, which then would enable a fast biodegradability. As such, ester moieties are well known to be cleaved easily by microorganisms through hydrolysis due to the presence of hydrolases, in particular lipases and esterases.

Accordingly, we became interested in inserting ester group moieties into a hydrocarbon network in a manner that still maintains the required lubrication properties while ensuring rapid biodegradation as well as a straightforward synthetic access utilizing well‐established industrially feasible catalysis technologies and biorenewable instead of fossil feedstocks [8, 9]. Note that, in contrast, today’s commercial lubricants are mostly produced from fossil feedstocks as raw materials. Our envisaged structures that address the issues of biodegradability and access from biobased feedstocks are shown in Scheme 1B [8, 9]. A further key criterion of the new lubricant product class is modularity. In other words, starting from the same types of raw materials and based on the same type of applied process technologies, the accessible final product should be able to be modified in a tailor‐made fashion so that the properties, e.g., viscosity, can be adjusted to meet the desired requirements accordingly.

**SCHEME 2:**
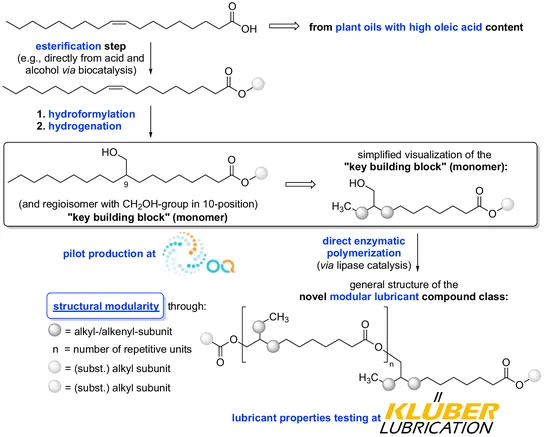
Synthetic concept for an efficient approach to the desired biobased and biodegradable target lubricant molecules, enabling favorable economics as well as structural flexibility.

Furthermore, from a retrosynthetic perspective such target molecules should be accessible in a few steps from readily available and cheap raw materials. The proposed retrosynthetic pathway is graphically shown in Scheme 2. Our concept has been based on oleic acid and a readily available alkyl ester, e.g., methyl ester, thereof as a cheap raw material. The initial steps consisted of a hydroformylation and hydrogenation, which furnish the needed hydroxy ester moiety for oligoester formation. At the same time, the presence of long and branched hydrocarbon structural units should ensure the required lubrication properties, thus making this compound a promising monomer for building up oligoesters and polyesters as lubricants. Hydroformylation is the worldwide largest applied homogeneous metal‐catalyzed process with a production volume exceeding 14 million tons [10], thus making it an ideal catalytic method for our purpose. Hydrogenation represents a further matured technology that is technically feasible for cheap bulk chemicals production [11]. In earlier work, we focused on the direct hydroformylation of oleic acid [12], while in our later academic–industrial collaboration with OQ Chemicals (Oberhausen, Germany; now: OXEA) hydroformylation of an alkyl oleate turned out to be more preferable and such processes were scaled‐up in this project [13]. The next step consisted in a tailor made and at the same time highly economical and sustainable conversion of the monomer to oligo‐ and polyesters thereof. Here we applied biocatalysis with an immobilized enzyme in a solvent‐free process, which also enabled us to adjust the chain length of the oligo‐ or polyester to the desired extent in a tailor‐made fashion [8, 9]. With these catalysis tools in hand, which also underlines the value of combining chemo‐ and biocatalysis when addressing challenging industrial syntheses, we could design a broad panel of such biobased oligo‐ and polyesters as lubricants in a modular fashion with one type of chemoenzymatic technology platform.

Next, the properties of such compounds for lubricant applications have been subject of our studies. Here, the modular synthetic concept enabled us to specifically adjust the pour point that is the lowest temperature at which an oil will flow when being cooled. The developed products showed the desired low pour points to avoid their solidification at low environmental temperatures, and the pour point can be decreased when increasing the degree of oligomerization [9]. For example, seven repetitive units then gave a favorable pour point of −18°C. Furthermore, the physical parameters viscosity and viscosity index also could be adjusted to be in an attractive range for lubricant applications. In addition, biodegradability of such biobased lubricant oils as a key criterion for marine applications was shown by means of the OECD 301 F test. A rapid biodegradation was observed for both a low‐viscosity product (viscosity at 40°C: 119.9 mm^2^ s^−1^) and a high‐viscosity product (viscosity at 40°C: 408.7 mm^2^ s^−1^), which can be rationalized by the presence of ester moieties in the oligomer as readily hydrolyzable groups in microbial degradation.

**SCHEME 3:**
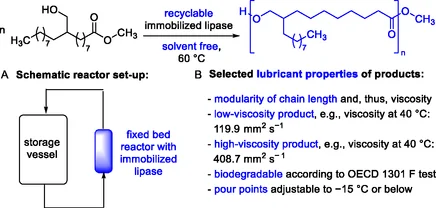
Biocatalytic oligomerization with efficient enzyme recycling through utilization of a heterogenized biocatalyst in a fixed bed reactor.

In order to make this process for synthesizing the lubricant oils technically feasible and more sustainable as well as cost‐efficient, recycling of the biocatalyst is mandatory due to the high price of the biocatalyst compared to the final lubricant product. Accordingly, we designed a semibatch process with the immobilized enzyme being embedded in a fixed‐bed reactor (Scheme 3) [14]. The monomer in the storage vessel as a “bubble storage tank” with a maximum capacity of 100 g is then pumped through this fixed‐bed reactor and the formed monomer/oligoester mixture is then pumped back to the storage tank until the desired chain length of the oligoester/polyester is reached. Note that, besides enabling a process with integrated separation of the product from the immobilized biocatalyst, this process is completely work‐up free since the final oligoester or polyester product can be simply taken from the storage tank as obtained without the need of any downstream‐processing. Avoiding any need for a solvent and the absence of any downstream‐processing unit operation steps then also result in a very low E‐factor of <0.15, which is in an excellent range indicating a high sustainability of the process [14].

The impact of enzyme recycling on economics is underlined by the fact that the overall biocatalyst loading is extremely low since in a recycling study no significant loss of activity was observed within 10 cycles. Assuming a loss of only 0.25% of the biocatalyst activity per cycle (as determined in a closely related study for another ester) [14] and taking into account literature‐reported estimated enzyme costs [15] resulted in calculated biocatalyst costs for this enzymatic oligomerization of less than 0.50 € per kg of the obtained lubricant oligomer [14].

A further key advantage of this method is the tailor‐made length of the oligoesters which can be adjusted by means of a lipase as a biocatalyst, thus enabling the access to lubricants with low viscosity (short‐chain oligoesters) and high viscosity (long‐chain oligoesters/polyesters). By means of this reactor, synthesis of a lubricant oil with desired properties such as a viscosity of 148.1 mm^2^ s^−1^ (40°C) and 18.9 mm^2^ s^−1^ (100°C), a viscosity index of 145 and a pour point of −12°C was demonstrated [14].

It should be added that this industrial–academic achievement on the design and efficient synthesis of these biodegradable de novo‐lubricants based on biorenewable feedstocks has been recently awarded in 2025 the sustainability prize (“*Nachhaltigkeitspreis*”) of the Environmental Foundation of the Eastwestphalian Economy (“*Umweltstiftung der ostwestfälischen Wirtschaft*”) [16].

## 3 Design of Biobased & Biodegradable Polyethylene (PE)‐Like Plastics and Process Development for their Syntheses

**SCHEME 4:**
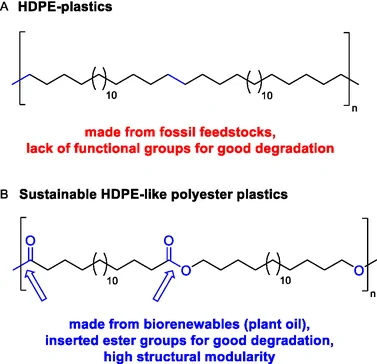
Conceptual design of biobased polyethylene (PE) alternatives to fossil feedstock‐based PE, which still contain the alkyl structures needed for PE‐like properties.

In the industrial segment of plastics, the lack of degradability of today’s PE products has been addressed also by means of the concept of introducing ester functionalities for enabling a subsequent cleavage of the polymer at the ester moieties through methanolysis. Such a development in the field of biobased and degradable polyesters derived from fatty acids was reported by the Mecking group in 2021 [17]. In this work, PE‐like polyesters were prepared which show similar properties as PE, but are much better degradable due to the presence of ester moieties and allow a depolymerization back to the monomer through ester cleavage. The structural concept of such polymers is shown in Scheme 4.

**SCHEME 5:**
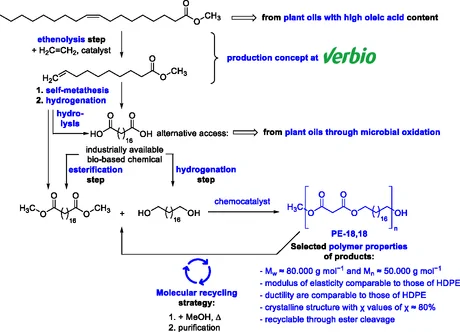
Synthetic concept for an efficient approach to the desired biobased PE‐like polymers, enabling favorable economics as well as structural flexibility.

The design of such biobased and recyclable polyesters is based on the utilization of plant oil feedstocks [17]. The polymerization of such monomers then leads to exactly the properties that are needed to fulfill the criteria of performance and degradability. The long alkyl chain fragments are the ones being also present in PE‐type polymers, while the ester moieties being introduced in a defined mode through the polymerization process of the designed monomers enable a subsequent recycling through methanolysis. The polymerization step has been performed in a classic fashion via polycondensation with the titanium alkoxide [Ti(O*n*Bu)_4_] as catalyst (Scheme 5) [17]. The overall synthetic concept to access such polyesters is based on utilization of a C18 dimethylester and C18 diol derived from 1,18‐octadecanedioic acid, being accessible from plant or microalgae oils as biorenewable feedstocks. This C18 diacid as a starting material is a readily available chemical being technically accessible by microbial production or from oleic acid via metathesis and hydrogenation [18, 19]. It should be added that recently Verbio announced to build up a plant for producing methyl 9‐decenoate on a 32.000 tons scale [20], from which the C18 diacid and its esters are accessible by means of metathesis and hydrogenation.

For the preparation of the polyesters, a C18 dimethylester as well as a C18 diol were prepared from the C18 diacid as monomers [17]. The corresponding C18 dimethyl ester has been synthesized by esterification with methanol, and subsequent hydrogenation gave the C18 diol. Subsequent polycondensation of the C18 diester with the C18 diol then furnished the desired polyester, which has been termed polyester‐18,18 (PE‐18,18).

Furthermore, the Mecking group applied this concept of biobased PE‐like structures with so‐called “in‐chain functional groups as break points” to the polymer class of polycarbonates [17]. For the sake of completion it should be added that in previous work such polymers PE‐18,18 and PC‐18 were already prepared from other feedstocks [21, 22].

By means of their synthesized polymers, the Mecking group demonstrated that these compounds show PE‐like mechanical properties [17]. The polyester formation of PE‐18,18 via polycondensation of the C18 diester with the C18 diol in the presence of titanium‐alkoxide furnished a plastic compound with typically *M*_w_ ≈ 80.000 g mol^−1^ and *M*_*n*_ ≈ 50.000 g mol^−1^, which is comparable to the molecular weight data of commercially available high‐density polyethylene (HDPE). In addition, materials testing revealed similar properties as the ones of HDPE, and the Mecking group showed that modulus of elasticity and ductility are comparable to those of HDPE samples [17]. It is noteworthy that in spite of the presence of such ester (or alternatively carbonate) moieties, a crystalline structure as in case of HDPE is achieved, which has been shown by means of wide angle X‐ray scattering (WAXS) diffractograms. According to such WAXS data, the solid‐state structure of these polymers is dominated by crystallization of aligned hydrocarbon segments. With *χ*‐values of *χ* ≈ 80% the degrees of crystallinity (*χ*) turned out to be essentially the same as the one of HDPE samples. However, the crystallization points of PE‐18,18 are below the ones of HDPE, which has been rationalized by an “energy penalty” due to the presence of ester moieties within the crystalline segments.

The chemical recycling of these materials has been done by solvolysis, and when using PE‐18,18 in a noncatalyzed depolymerization in MeOH, a mixture of the monomers C18 diester and C18 diol was obtained [17]. This methanolysis proceeds in a catalyst‐free fashion at 150°C and leads to full depolymerization within 12 h at a polymer loading of 40 g/L. Note that in comparison chemical recycling of HDPE materials proceeds at much higher temperatures through pyrolysis above 500°C. Furthermore, the methanolysis also proceeds efficiently in case that the HDPE‐like polyester material has been blended with an HDPE sample, which remained unchanged after the solvolysis process. Although the obtained mixture consisting of C18 dimethylester and C18 diol as two high‐boiling components is difficult to be separated, it can be used after removal of MeOH without further product isolation for a re‐polymerization to furnish again PE‐18,18 with similar material properties in a titanium‐catalyzed polycondensation reaction [17]. As such material tests, tensile testing of injection‐molded and 3D‐printed specimens were conducted.

**SCHEME 6:**
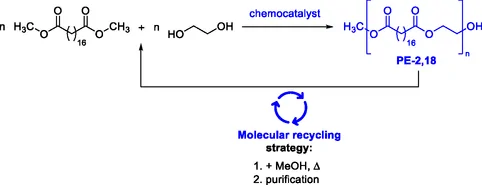
Process concept for formation and recycling of PE‐like polymer from the C18 diacid and the shortened diol ethylene glycol as biorenewable feedstock‐based monomers.

In a further work, the Mecking group reported a similar, also biobased polyester based on the C18 dimethyl ester and a shortened diol, which turned out to be advantageous, as monomers (Scheme 6) [23]. As such a shortened diol, ethylene glycol has been utilized. The resulting polyester materials also show a PE‐like solid‐state structure, tensile properties being similar to HDPE and crystallinity with a high melting point of 96°C. However, this polyester‐2,18 (PE‐2,18) turned out to be completely degraded by hydrolysis in the presence of isolated enzymes. Furthermore, under standardized composting conditions the material is biodegraded within 2 months. The improved biodegradation was shown to be related to the shorter “diol‐based subunit” in the polyester structure. As in case of the polyester‐18,18, also this polyester‐2,18 can be depolymerized chemically by means of solvolysis with MeOH in an efficient way, thus enabling again a “closed‐loop recycling” [23].

## 4 Conclusion

In conclusion, this “Perspective”‐article describes recent developments in the field of biobased and biodegradable oligomers and polymers for applications in the lubricant and plastics product segments, which are accessible from unsaturated fatty acids as biobased and economically attractive feedstocks. Therein, also the various criteria that have to be fulfilled along the way from plant feedstocks over monomers to the final oligo‐ and polyester products are discussed. Within an industrial–academic collaboration, oleates have been converted into hydroxy‐methylated stearates using the well‐established industrial technologies hydroformylation and hydrogenation. Subsequent solvent‐free enzymatic transesterification then furnished novel types of oligoesters or polyesters as lubricants that are biobased, ensure rapid biodegradation, and show needed performance data. Furthermore, when starting from C18 diester being also derived from fatty acid sources, polyesters have been designed through polycondensation with biobased diols such as C18 diol or ethylene glycol. The resulting polyesters showed material properties being comparable to high HDPE‐type materials and enabled additionally a closed‐loop recycling due to their proven degradability properties.

Key success factors in both projects [8, 9, 13, 14, 16, 17, 23] were the integration and consideration of criteria from all segments “along the value chain” (shown in Figure 1), ranging from the choice of the biobased feedstock, the synthetic access to the monomer, the design of the tailor‐made oligo‐ or polyester and their properties addressing performance as well as sustainability issues such as biodegradability or recyclability. In addition, combining the expertises from academia and industry turned out to be fruitful as demonstrated in the biobased lubricants project with participation of the lubricant producer Klüber Lubrication München (Germany) as well as the bulk chemicals producer OQ Chemicals (Oberhausen, Germany; now: OXEA) with their hydroformylation and hydrogenation catalysis core technologies [8, 9, 13, 14, 16].

It should be added that besides these two selected examples, in recent years numerous other approaches to polyesters starting from unsaturated fatty acid‐based biorenewable feedstocks were reported. Such polyesters are, for example, based on epoxidized fatty acids (and a reaction with a cyclic anhydride component) [24], (*R*)‐10‐hydroxystearic acid methyl ester (obtained within a chemoenzymatic process) [25], and fatty acid dimethyl esters (obtained through a catalytic dehydrogenation of a saturated fatty acid with O_2_, esterification and dimerization with dithiols within a thia‐Michael addition) [26] as different monomers. Accordingly, many exciting perspectives for future design and applications of tailor‐made biobased oligo‐ and polyesters from fatty acid‐based raw materials arise. It can be expected that starting from these biorenewable feedstocks, a broad range of oligo‐ and polyesters, fulfilling challenging performance as well as sustainability criteria will be accessible on scale in efficient and economical fashion. At the same time such product designs and process developments are complex and highly interdisciplinary tasks (with some of the criteria being shown in Figure 1). Thus, bundling competencies from different scientific fields, such as, e.g., organic synthesis with biorenewables and polymer applications, as well as the set‐up of joint academic and industrial project teams and networks appear to be among the success factors also for such future developments in this research area.

## Disclaimer

The opinions expressed in this publication are the view of the author(s) and do not necessarily reflect the opinions or views of ChemSusChem, the Publisher, ChemistryEurope, or the affiliated editors.

## Funding

This study was supported by the German Ministry of Food and Agriculture (BMEL) and the Fachagentur für Nachwachsende Rohstoffe (FNR) (grant no. 22026218).

## Conflicts of Interest

The author declares that he has no competing interests. However, for the sake of completeness it should be added that he is co‐inventor of a filed patent application (cited as reference [8]) that has led to patents being granted in various countries, covering findings described in the first part of this article about biobased and biodegradable lubricants.
